# Decoding retinitis pigmentosa: molecular targets and therapy with focus on pre-mRNA splicing

**DOI:** 10.1007/s00018-025-05987-0

**Published:** 2025-11-28

**Authors:** Poulami Banik, David Staněk

**Affiliations:** Institute of Molecular Genetics of the Czech Academy of Sciences, Videnska 1083, Prague, Czech Republic

**Keywords:** Splicing, Retinitis pigmentosa, Therapeutics, Models, Molecular mechanisms

## Abstract

Retinitis pigmentosa (RP) is the most common cause of inherited blindness, with mutations in splicing factors playing a significant role in its pathogenesis. Many scientists have been puzzled by the fact that mutations in several key spliceosomal components have such a confined effect on the retina. In this review, we summarize findings gained from studies using cell culture, animal models, and retinal organoids to better understand the molecular mechanisms underlying the tissue specificity of splicing factor dysfunction to retinal degeneration. Although RP currently has no definitive cure, recent advances in gene therapy, antisense oligonucleotides, and cell transplantation are opening new therapeutic approaches to slow disease progression and preserve retinal function. We also discuss the strengths and challenges of current strategies and point to the critical improvements required for their successful clinical application.

## Introduction

**Fig. 1:**
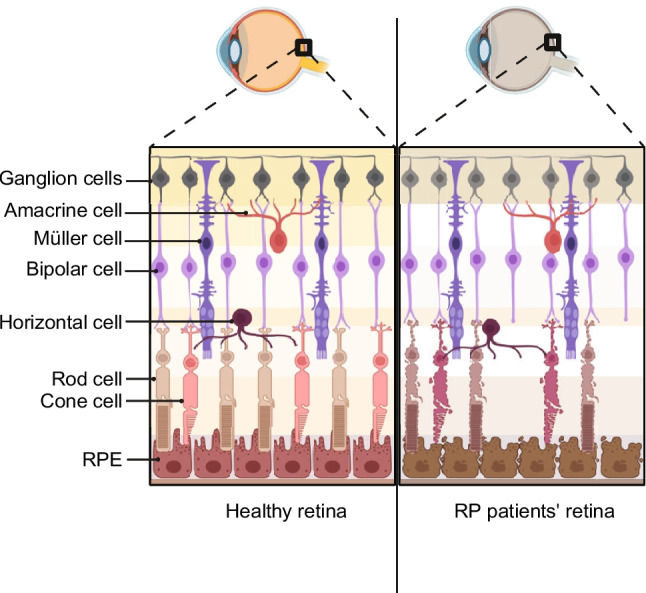
Comparison of retinal structure in a healthy eye and in RP. (Left) Schematic illustration of a normal retina showing organized layers of retinal neurons and supporting cells, including ganglion cells, amacrine cells, Müller cells , bipolar cells, horizontal cells, rod photoreceptors, cone photoreceptors, and the retinal pigment epithelium (RPE). Photoreceptors are abundant and form intact outer and inner segment layers. (Right) Schematic illustration of a retina affected by RP. Progressive photoreceptor degeneration leads to thinning and disorganization of the outer nuclear layer, loss of rod and cone cells, and secondary changes in the inner retinal layers and RPE. These structural defects underline the progressive vision loss observed in RP patients. Created in BioRender. Stanek, D. (2025) https://BioRender.com/co6hwir

Retinitis pigmentosa (RP) is an inherited retinal dystrophy in humans primarily caused by the degeneration of photoreceptors (Fig. 1). Its prevalence is approximately 1 in 4,000, making it the most common cause of inherited blindness [1, 2]. RP is classified into two types: if the retina is the only affected tissue, it is called non-syndromic RP. When other organs such as the ear, kidney, or brain are affected, this condition is referred to as syndromic RP [3, 4].

**Fig. 2:**
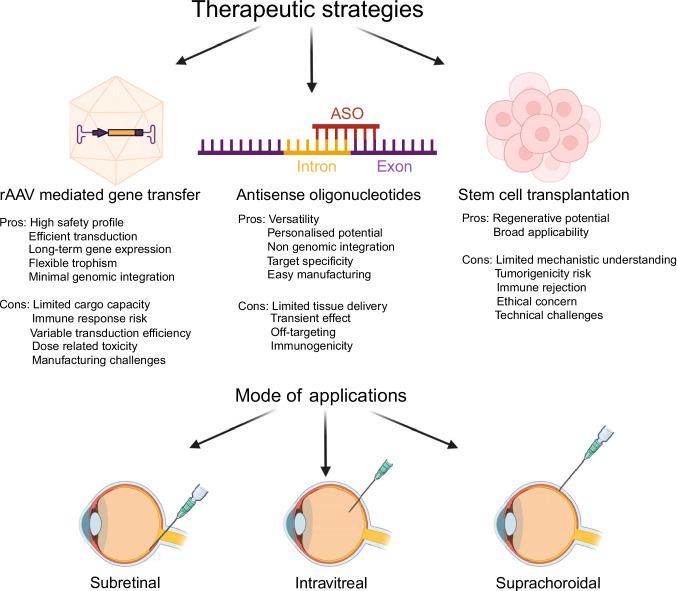
Overview of therapeutic strategies for RP. This summary covers key methods, including rAAV-mediated gene transfer, antisense oligonucleotides (ASOs), and stem cell transplantation, highlighting their primary benefits and limitations. rAAV supports long-term gene expression but is constrained by cargo capacity and immune responses. ASOs allow for adaptable, non-genetic regulation of splicing but face challenges in delivery and potential toxicity. Stem cell therapies offer regenerative possibilities but also involve risks, including tumor development, immune rejection, and technical hurdles. The common ocular delivery routes- subretinal, intravitreal, and suprachoroidal- are depicted. Created in BioRender. Stanek, D. (2025) https://BioRender.com/kum7nt8

RP is genetically a highly heterogeneous disease, with mutations identified in over a hundred different genes that can trigger the condition (retnet.org). In about half of the cases of non-syndromic RP, a single occurrence of RP has been reported within a family. In cases where family inheritance is clearly documented, non-syndromic RP is most often inherited in an autosomal dominant (adRP) manner (15–25% of cases), followed by autosomal recessive inheritance (5–20%), and finally, X-linked inheritance (~ 10% of cases) [5]. The majority of RP mutations affect genes directly involved in light detection, the visual cycle, and the morphology and metabolism of retinal cells (Figs. 1 and 2).

This review focuses on pathogenic variants in general splicing factors, which are the second most common cause of adRP. Unlike mutations in genes involved in retinal function, where the connection to the retina is obvious, defects in spliceosomal proteins should affect all cells in the body. This makes the confined defects to the retina particularly enigmatic. Despite 25 years of research, the reason why the retina is so sensitive to spliceosome malfunctions remains unclear. The pathogenic variants primarily target protein components of the U4/U6•U5 tri-snRNP, a key part of the spliceosome (see [6] for review). Recently, several mutations in U4 and U6 snRNAs were also reported as a cause of adRP [7]. In contrast, mutations in two other spliceosomal snRNPs—U1 and U2—have not been linked to RP, suggesting that the retina is specifically vulnerable to defects in the tri-snRNP. It should be noted that snRNPs forming the tri-snRNP are 10–100 times less abundant than U2 and U1, respectively [8]. However, mutations in tri-snRNP-specific proteins are not exclusive to retinopathies. For example, haploinsufficiency of EFTUD2 causes mandibulofacial dysostosis with microcephaly [9], and downregulation of TXNL4A protein results in defective facial development and hearing loss [10]. Somatic mutations in *PRPF8* have been associated with myeloid neoplasms [11], and overexpression of PRPF8 increases the aggressiveness of hepatocellular carcinoma [12].

### Animal models to study splicing-related RP

The reasons why various mutations in different components of the same spliceosomal complex cause different disorders are still unclear. To address this issue, many animal models have been developed to study the effects of splicing mutations on retinal cells. Early studies focused on expressing mutated proteins in cultured cells. These experiments showed how mutations in splicing proteins impact retinal function but provided little insight into why retinal cells are particularly vulnerable to these mutations (reviewed in [6, 16]). Therefore, more complex animal models were needed to study RP. Two studies reported that downregulation and mutation of *PRPF8* and *SNRNP200* in *C. elegans* led to developmental defects but did not provide enough evidence regarding retina-specific phenotypes in humans [17, 18]. Similarly, expression of nine RP variants of *D. melanogaster* PRPF8 ortholog caused developmental defects, and two pathogenic mutations (dPrp8S > F/hPRPF8^S2178F^ and dPrp8H > R/hPRPF8^H3169R^) negatively affected eye morphology [19]. The relatively low costs of maintaining non-vertebrate models, along with clear phenotypes, make these models ideal for large-scale screens to identify genetic or chemical modulators of splicing factor depletion or mutation.

Research on vertebrate models with eye chambers similar to humans has established many retinal degeneration models in *D. rerio*, but few have focused on splicing factors. Early zebrafish studies relied on downregulating splicing factors with mixed results. Reduced expression of PRPF3, for example, did not cause visible eye abnormalities [20]; however, silencing of *Prpf31, Prpf4, *and* Snrnp200* negatively affected photoreceptor structure and visual function [21, 22]. Consistent with this, a mutation in *Prpf4* caused cell death and neurodevelopmental defects, likely by disrupting the Wnt signaling pathway [23]. Expression of RP-linked *Prpf31* mutations in zebrafish showed that C-terminally deleted PRPF31, called SP117 (c.769insA in humans), and AD5 (c.1115_1125 del in humans) variants led to protein destabilization and mislocalization in the cytoplasm [24]. Rapid degradation and cytoplasmic localization were also observed in the human PRPF31^A216P^ RP variant [25]. Additional studies revealed that PRPF31 depletion affects the splicing of exons with weak splice sites and reduces the viability and proliferation of zebrafish retinal progenitor cells [26]. The importance of the splicing machinery for zebrafish retina development was further demonstrated by depleting SF3B4, a protein specific to U2 snRNP [27]. However, it is important to note that SF3B4 haploinsufficiency in humans is associated with Nager syndrome, an acrofacial dysostosis characterized by upper limb and facial-mandibular defects, rather than RP [28]. While the zebrafish model successfully mimicked retinal degeneration caused by disruptions in the splicing machinery, it did not reveal a consistent molecular mechanism underlying the disease.

Finally, RP has been studied using a mouse model. Initial attempts involved deleting one allele of the *PRPF3* and *PRPF31* genes to test whether haploinsufficiency of splicing factors can cause retinal dystrophy. While early reports did not observe any retinal phenotype [20, 29], later studies indicated degeneration of the retinal pigment epithelium (RPE) in one-year-old mice [30]. The same study reported RPE defects after expressing protein variants that mimic RP-linked substitutions PRPF3^T494M^ or PRPF8^H2309P^ [30]. These data suggest that the RPE is the primary target of RP mutations, at least in mice, and that impaired phagocytosis underlies the defect [31]. Further research revealed artificial aggregation of PRPF31^A216P^ in the cytoplasm of RPE [32], which aligns with previous studies on zebrafish and human cell cultures [24, 25]. However, analysis of two RP-mimicking mutations in the *PRPF8* gene (Y2334N substitution and a C-terminal extension E2331VfsX15) did not show any retinal phenotype, but both Prpf8 mutants, when expressed as homozygotes, showed significant degeneration of granule neurons in the cerebellum [33].

Thus, despite new findings and advances in our understanding of RP, animal models have not (yet) provided a definitive answer regarding the tissue specificity and molecular mechanisms of RP caused by splicing factor mutations.

### Retinal organoids

Animal models have offered valuable insights into the pathophysiology of RP. However, they have not been able to reproduce all the disease phenotypes seen in humans. This is mainly because the homologous genes in animal models differ in their expression, and the retina's physiology in mice or zebrafish is not the same as in humans [34]. In some cases, the mutation phenotype does not even appear in the retina [33]. To overcome these limitations, 3D retinal organoids (RO) derived from human induced pluripotent stem cells (iPSCs) have shown promise as an in vitro alternative.

Human iPSCs can be reprogrammed from patient somatic cells and hold significant potential to differentiate into various cell types important for RP studies, such as RPE, retinal progenitor cells, retinal ganglion cells, and RO [35–37]). ROs resemble the human retina and contain retina-specific cells, including rods, cones, Müller cells, amacrine cells, bipolar cells, and ganglionic cells. Therefore, they have been used to study eye development as well as RP progression (e.g. [38–40]). Additionally, ROs can be produced at scale and do not raise ethical concerns. Since their inception in 2011 from mouse embryonic stem cells [41], and later from human embryonic stem cells in 2012 [42], and human iPSC in 2014 [37], various groups have used this model to study RP and other retinopathies. To date, RP-RO models have been used to investigate several RP mutations. Mutations in PDE6B and CRB1 recapitulate photoreceptor defects [43, 44], and an RO with class 3 RHO mutations showed mislocalization of rod photoreceptors and reduced rhodopsin expression [45]. Photoreceptor differentiation and maturation were impaired in RP2-deficient ROs, leading to photoreceptor cell death [46]. ROs derived from mutated USH2A exhibited early developmental defects characteristic of RP [47]. ROs from patient-derived iPSCs, with the RPGR mutation corrected via CRISPR-Cas9, restored the structure and function of the retinal organoids [48]. RO containing an ARL3 mutation revealed mislocalization of several protein factors, indicating defective protein transport in photoreceptors [40]. With few exceptions, human ROs can mimic disease phenotypes and serve as effective models for studying various retinopathies.

ROs were also used to study RP-linked mutations in splicing factors. RPE and RO were differentiated from iPSCs derived from a patient carrying various PRPF31 mutations. Transcriptome analyses of RPE and RO differentiated from the patient’s iPSC revealed splicing defects in key components of the spliceosome, as well as in genes enriched in cell junctions and in microtubular and centriole organization [13, 49]. This was associated with morphological changes, including shorter microvilli and cilia, non-polarized cells, loss of cellular junctions, and defects in phagocytosis [13]. Reduced expression of PRPF31 caused defects in RPE and RO, which were rescued by overexpression of PRPF31, indicating that low levels of PRPF31 protein cause RP phenotypes [50, 51]. Recently, iPSCs from a patient carrying the p.H2309P mutation in PRPF8 were used to produce RPE and RO. The mutant RPE cells appeared unpolarized and exhibited ciliary defects, while the ROs showed degeneration and a reduced number of photoreceptors [52].

ROs are not only proven useful in characterizing RP; they have also been used to test various therapeutic strategies. Patient-derived organoids with a monoallelic mutation in RHO-CNV were targeted with a small molecule, photoregulin3, which restored RHO localization in the inner and outer segments of rod photoreceptors [53]. The RP-RO model has also been extensively used for testing gene therapies (see below) [46, 54–56]. Recently, stem cell replacement therapies have gained popularity for treating retinal dystrophies, and ROs play a vital role in this process (see below).

Using RO to model and treat retinal diseases involves several challenges. Even those derived from healthy cells exhibit limited light responsiveness because of underdeveloped outer segment discs [37]. The contact between neural retina and RPE, which are responsible for supporting photoreceptors, is missing in RO. Additionally, retinal ganglion cells tend to degenerate within organoids owing to the absence of connections to brain targets, which hinders the formation of functional circuits. Further complications include high cellular heterogeneity, uneven maturation, and inconsistent lamination, making comparisons and therapeutic evaluations difficult [57]. Finally, it takes at least 6 month of RO culture to obtain fully mature photoreceptors, making them a suboptimal model for testing potential therapies.

### Molecular mechanism of RP

Many researchers are trying to understand the molecular mechanisms behind why mutations in general splicing factors lead to tissue-specific phenotypes. This effort is driven by curiosity to understand the principles of the disease, but it is also essential for finding a cure. There are two key questions in the field: 1. Why is the retina particularly vulnerable to splicing factor mutations? 2. How do mutations in splicing factors trigger retinal degeneration?

High metabolic activity and constant renewal of photoreceptor segments suggest that photoreceptors have a high demand for splicing factors, making any disruption in the splicing machinery critical for them. This idea is supported by findings that the retina expresses higher amounts of spliceosomal snRNAs than other tested tissues and that mutations in splicing proteins reduce the levels of splicing-competent complexes, leading to splicing defects [13, 52, 58]. However, the expression of PRPF31 is higher in RPE than in neuroretina [32]. Moreover, this hypothesis does not explain why RP is typically diagnosed in the second or third decades of life. Recent data from mouse models and human retinas shed light on this discrepancy. First, reduced expression of PRPF31 mRNA correlates with age in humans [59, 60]. Additionally, decreased expression of splicing factors was observed in the wild-type mouse cerebellum and retina, suggesting that a physiological, age-related decline in splicing proteins may make these tissues more vulnerable to mutations in spliceosomal proteins [33]. Therefore, it appears that photoreceptors do not significantly overexpress tri-snRNP-specific proteins, and the levels of these proteins and tri-snRNP complexes may be close to a functional threshold. Variations in PRPF31 expression have been proposed to explain the incomplete penetrance of PRPF31 mutations and have been linked to the expression of CNOT3, a transcription factor that negatively regulates PRPF31 transcription [61, 62]. However, recent studies challenge this view, finding no correlation between PRPF31 and CNOT3 expression [59, 60]. Instead, another study linked PRPF31 expression to the number of MSR1 repeat elements in its promoter region [59, 60, 63]. Incomplete penetrance has also been reported for mutations in the *PRPF8* gene, though the mechanisms remain unexplored [64]. Based on available data, the abundance of splicing factors, especially splicing-competent tri-snRNPs, appears to be a key factor that makes retinal cells susceptible to mutations in spliceosomal proteins. This includes mutations that decrease the expression of specific splicing proteins as well as those that impair tri-snRNP function. A reduction in one tri-snRNP protein often results in decreased levels of other tri-snRNP components, effectively lowering overall tri-snRNP levels [14, 49, 58]. Combined with the natural age-related decline in spliceosomal proteins within the retina, this vulnerability makes photoreceptors particularly susceptible to mutations in tri-snRNP components.

Many studies have been conducted to understand the molecular mechanisms behind defects linked to mutations in splicing factors. The suspicious accumulation of RP mutations in tri-snRNP components suggests that retina cells are particularly vulnerable to defects in this complex, while mutations in U1 and U2 snRNP cause different disorders [65]. Could there be a retina-specific, splicing-independent role of tri-snRNP in photoreceptors? So far, there is no clear evidence supporting this idea, and this hypothesis does not explain mutations in *PRPF8* and *SNRNP200*, where the mutated proteins are normally incorporated into the tri-snRNP and the spliceosome [14, 15, 52, 66]. Some substitutions impact the protein's function and likely affect proper folding [67, 68]. The ongoing activation of the unfolded protein response may then activate persistent stress leading to apoptosis. However, this model does not clarify how RP-linked mutations that reduce transcription or cause mRNA degradation result in photoreceptor death, since no misfolded proteins are produced in these cases. A search for retina-specific isoforms of splicing factors yielded no positive results [69], suggesting that the main affected function of RP mutations relates to pre-mRNA splicing, and most research has focused on this aspect.

Initial studies using retinal gene-derived splicing reporters showed that mutations in splicing factors affect the splicing of several retina-specific genes, such as *RHO*, *PROMININ1*, and *PAX6* [15, 70–72]. Similarly, mis-splicing of phototransduction genes was observed in human organotypic retinal cultures in response to reduced PRPF31 expression [73]. Efficiency of intron removal and changes in alternative splicing were noted in lymphoblasts from various RP patients with mutations in splicing proteins [58, 74]. Comparing splicing defects in two mouse PRPF8 models showed no overlap in protein-coding genes but revealed common mis-splicing of circRNAs, which are highly expressed in neural and retinal tissues [33]. However, it remains unclear whether changes in circRNA production are markers of defective splicing machinery or if circRNA misexpression directly impacts neuron survival. Two studies analyzing RNA splicing in RPE and retinal organoids derived from patient iPSC highlighted alterations in the splicing of genes essential for cilia formation and maintenance. Consistently, they observed defects in cilia morphology in RPE and photoreceptors [13, 52, 59]. A detailed analysis showed that introns with weak 5' splice sites are especially susceptible to mutations in the splicing machinery [52]. A recent study supported the splicing-centric view of the molecular cause of RP. The study showed that two mutations in the helicase domain of SNRNP200 impair its U4/U6 snRNA unwinding activity and spliceosome activation, and mutations in PRPF8 affect splicing kinetics [75]. In addition, inhibition of splicing by the splicing inhibitor E7107 caused loss of vision in two out of 26 tested individuals [76]. However, despite significant efforts, it remains unclear which gene mis-splicing causes retinal degeneration though genes involved in cilia structure and visual signal transduction are strong candidates. Although no single definitive mis-spliced “killer” transcript has been identified, changes in IFT122 (essential for intraflagellar transport) splicing and circRNA misexpression have been observed in different pathogenic variants of splicing factors indicating that a common set of sensitive genes can exist [13, 33, 77].

We would like to conclude the current research on why mutations in splicing proteins cause RP. The unifying principles are the age-related decline of several key components of the splicing machinery in connection with pathogenic mutations in tri-snRNP factors, creating a lethal combination. Reduced spliceosome activity affects the splicing of numerous genes, including those involved in cilia structure and visual signal transduction. This creates a downward spiral that ends in retinal cell death. Consistently, many trials attempting to treat RP caused by pathogenic variants in splicing proteins are focusing on augmentation of splicing protein expression in the retina.

### Potential therapies for RP

Various approaches have been tested to combat retinal disorders, including gene replacement therapy using recombinant adeno-associated virus (rAAV), antisense oligonucleotides (ASO), siRNAs, CRISPR-Cas9 genome editing, and stem cell replacement therapy by introducing retinal cells into the patient's eye (Fig. 2). Additionally, various small molecules have been explored for treatment or slowing RP progression. These trials involve the antioxidant N-acetylcysteine, vitamin A in combination with lutein, docosahexaenoic acid, vitamin E, valproic acid, analogues of folic acid, or more specific small molecules EA2353, which aim to specifically activate retinal stem and progenitor cells (clinicaltrials.gov). Details of the selected therapeutic strategies are discussed below.

### Gene therapy

Research has advanced significantly over the past 30 years in rescuing mutated genes by introducing functional gene products. This technology has proven effective in treating several previously untreatable inherited diseases. Unlike traditional drugs that usually target proteins, these nucleic acid-based drugs aim to modify gene expression. This approach has been shown to be safe, specific, and efficient, leading the European Medicines Agency (EMA) and the U.S. Food and Drug Administration (FDA) to approve several gene therapy treatments, with many more in clinical trials. Gene therapy offers additional benefits for retinal dystrophies because the eye is an immune-privileged organ with a low immune response against viral vectors [78]. Moreover, the ease of administration and the small, confined volume of the eye make it an ideal target for gene therapies.

### rAAV-mediated gene augmentation

Viral vectors such as adenovirus, adeno-associated virus (AAV), lentivirus, and herpes simplex virus are used as carriers for targeted gene therapy because they offer higher transduction efficiency and specificity to a particular cell type compared to non-viral systems [79]. Among these viral vectors, AAV is the most preferred transgene delivery system by the FDA and EMA due to its non-pathogenic nature, ability to achieve long-term transgene expression without integrating into the host genome, broad cellular tropism, and high transduction efficiency [79]. Wild-type AAV consists of an icosahedral capsid made of 60 protein subunits that encase 4.7 kb of ssDNA. The genome is flanked by 145 bp inverted repeats that serve as the origin of replication and provide packaging signals. In recombinant AAV (rAAV), the viral genes are replaced with a transgene expression cassette, with only the inverted repeats retained. This enables rAAV to package and deliver 4.7 kb of foreign DNA into target cells. Different AAV serotypes vary in their capsid protein composition, influencing their tissue tropism and their ability to evade pre-existing antibodies [79].

The earliest attempt to transduce retinal cells in mice involved subretinal injection with an rAAV encoding the lacZ reporter [80]. Later, this approach was used in animal models to rescue various mutations causing retinal dystrophies in genes such as *PDEB* [81], *PRPH2* [82, 83], *MERTK* [84], *RHO* [85], *CNGB1* [86], *SPATA7* [87], *RP2* [88], *PDE6A* [89], and *PDE6B* [90]. Transducing rAAV-encoded proteins such as CNTF and GDNF in RP gene knockout mouse models provided promising results and improved photoreceptor survival [91–93]. Similarly, overexpression of the transcription factor NR2E3 increased *RHO* gene expression, slowed disease progression, and reduced photoreceptor loss in the RHO^P23H^ mouse model [94]. Additionally, rAAV gene therapy has been used to transduce a promoter that rescues both rod and cone degeneration in cases of AIPL mutation [95].

However, it should be acknowledged that despite many trials testing gene therapy-based approaches, Luxturna is the only FDA-approved gene therapy treatment for retinal dystrophy caused by mutations in the *RPE65* gene. This gene encodes a key enzyme that converts all-trans retinyl ester to 11-cis retinol. A functional copy of the *RPE65* gene is delivered using the adeno-associated viral vector serotype 2 (AAV2), which restores the visual cycle [96]. In a 2015 study, *RHO* mRNA mutation was rescued by Spliceosome-Mediated RNA Trans-splicing (SMaRT), a gene therapy technology that repairs mutated mRNA transcripts by using pre-trans-splicing molecules (PTMs) to replace mutated exons with correct sequences. In vivo, rAAV-mediated transduction of PTMs showed a trans-splicing rate of 9-22.5.5% [97]. Knockdown and replacement therapy have also been achieved with a single rAAV vector packaging shRNA to degrade the mutated gene and replace it with the correct cDNA in RHO adRP [98]. A similar approach has been used to express a mirtron in RHO knock-in mouse models [99]. With the advancement of stem cell models, iPSC and iPSC-derived RPE and RO have been adapted to test rAAV-mediated gene augmentation. *PRPF31*^+/-^ iPSC-derived RPE demonstrated the pathological defects associated with RP, which were partially rescued by transducing the cells with rAAV-PRPF31 [50, 55]. RP2 knockout ROs and patient-derived mutated RP2 ROs were transduced with RP2-rAAV, which rescued outer nuclear layer thinning and restored rhodopsin expression [46]. However, it is important to keep in mind the risks associated with virus-based delivery treatments. Although rAAV is considered a safe delivery system, there have been reports of viral genome integration in the liver and heart [100]. The use of genome editing tools, particularly CRISPR-induced double-strand breaks, has been associated with increased AAV genome integration in animal models, raising additional safety concerns [101].

### Modulation of gene expression by antisense oligonucleotides

Antisense oligonucleotides (ASOs) are 13-30 nucleotide-long synthetic nucleic acids that are emerging as powerful tools to regulate gene expression. ASOs bind to pre-mRNA or mRNA through standard Watson-Crick base pairing and influence gene expression by affecting processes such as pre-mRNA splicing, blocking translation, or promoting RNA degradation. They are heavily chemically modified to resist nuclease-mediated degradation and to improve their binding specificity for target sequences [102, 103]. ASOs can be categorized into different types based on their functions.

Gapmer ASOs or siRNAs are used to decrease gene expression. Gapmers are single-stranded oligodeoxynucleotides with modified bases at the 3’ and 5’ ends, which protect them from exonuclease degradation. Upon binding to the target RNA sequence, a gapmer forms a DNA:RNA hybrid that is recognized and cleaved by RNase H. siRNAs, on the other hand, are double-stranded RNA molecules that utilize the endogenous RNA interference pathway to degrade transcripts. Although siRNAs are more effective than gapmers at reducing gene expression [104], the RISC complex can tolerate a few mismatches and may potentially silence off-target genes. Furthermore, the sense passenger strand of the siRNA duplex can also be loaded into the RISC complex, which further increases the likelihood of affecting unrelated genes.

Single-stranded ASOs are also used to restore or modify pre-mRNA splicing. The ASOs are designed to bind regulatory elements or directly target the splice sites, preventing the interaction of splicing factors and regulators with the targeted sequences. Alternatively, ASOs can alter the secondary structure around splice sites, thereby changing their visibility to the splicing machinery. Finally, ASOs can also increase mRNA translation by sterically blocking the binding of translation repressors in the non-coding 5’UTR [105]. Due to their compact size and modifications, ASOs can be administered directly via subretinal or intravitreal injections without the need for a carrier.

The first generation of ASOs contained a modified phosphate backbone, where the non-bridging oxygen was replaced by sulphur, making ASOs resistant to nucleases. Currently used ASOs also include modifications at the 2’ hydroxy group in the ribose, such as 2’-O-methyl, 2’-O-methoxyethyl, 2’-aminopropyl, or 2’-fluoro [106]. The ASO chemistry must be carefully selected because different modifications affect ASO stability, efficacy, and toxicity [107]. Despite their successful application in treating several genetic diseases, there are no approved ASO drugs on the market for RP. However, several potential ASO-based approaches are under investigation. For example, ASO was tested to prevent the inclusion of a cryptic exon inserted into mRNA in patients with intronic mutations in the USH2A gene [108]. The two most common mutations in the *USH2A* gene occur in exon 13. Morpholino-induced skipping of exon 13 by ASO QR-421a (ProQR Therapeutics NV) was used to produce a functional protein lacking the damaged exon, which restored retinal function [109]. This treatment is now in phase II/III clinical trials [110]. ASO has also shown positive results in rescuing splice defects in a USH3A mouse model with a *CLRN1* mutation [111]. *RHO* and *PRPF31* are the most mutated genes associated with adRP. ASO specifically targeting and degrading rhodopsin with the p.P23H mutation has also been tested [112]. Another study targeted mutations in exon 12 of *PRPF31*, which often cause a shift of the reading frame and destabilize the mutated mRNA. ASO-mediated skipping of exon 12 restored the open reading frame and increased *PRPF31* mRNA production in fibroblasts derived from RP patients [113]. Most mutations in *PRPF31* lead to mRNA or protein degradation. An alternative approach aims to increase expression from a healthy *PRPF31* allele using an ASO that downregulates the negative transcription regulator CNOT3 [62].

ASO therapies face several major limitations that restrict their broader use. Effective delivery remains a significant obstacle, as only certain tissues like the retina, liver, and central nervous system currently achieve sufficient ASO uptake. The level of target modulation required for therapeutic benefit is often unclear, and excessive amount might cause harm [104]. Identifying the dose–response relationship and the duration of splice correction is therefore essential for choosing the best treatment schedule and determining if long-term benefits overcome the side-effects. Individual genetic differences can modify ASO–target interactions, affecting pharmacokinetics and pharmacodynamics; therefore, even minor nucleotide changes should be carefully evaluated for their effectiveness and target response [114]. Additionally, ASOs cannot treat all mutation types, particularly canonical splice-site variants and achieving allele-specific targeting in dominant disorders is challenging. Finally, ASOs are ineffective after significant cell loss has occurred, underscoring the need for early intervention.

### Cell transplantation therapy

The eye is an ideal organ for cell transplantation because it requires only a small number of cells to restore vision and has partial immune privilege that minimizes the risk of graft rejection [78]. Therefore, cell transplantation therapy is a popular approach to replace degenerated photoreceptors in patients with inherited retinal dystrophy. Current trials use various cell sources, including in vitro expanded neural progenitor cells, retinal progenitor cells, ESC or iPSC, mesenchymal stem cells, and bone marrow-derived stem cells, which are transplanted into patients (reviewed in [115]). Since these cells can self-renew and differentiate in multiple directions, successfully transplanted cells should differentiate into photoreceptors, integrate into the retina, and form synaptic connections with other retinal cells to restore vision. Additionally, the secretion of trophic factors from retinal precursors should help protect the retina from degeneration [116]. Initial experiments were conducted in mouse models of retinal dystrophy (reviewed in detail in [117]). Here are some key findings from transplantation studies: photoreceptor precursors can be successfully transplanted into the eye but the developmental stage of donor cells is a crucial factor for successful transplantation [118–120]. It has been shown that subretinally injected retinal progenitor cells mature into photoreceptors in the outer nuclear layer but the immune modulation is important for the long-term survival of transplanted cells in the retina [118, 121]. Given the success in mouse models, several human clinical trials have been conducted or are ongoing (Table 1) and [122].

**Table 1:** Cell transplantation therapies

Clinical trials(https://clinicaltrials.gov/)					
Number	Cell Type	Phase	No. of Patients	Administration Method	Status
NCT05800301	WJ-MSC	III	80	Injection in subtenon space of eye	Completed
NCT05712148	MSC(spheroid)	I/II	15	suprachoroidal implantation	Completed
NCT04925687	BMSC	I	4	intravitreal injection	Completed
NCT04604899	RPC	II	30	Intravitreal Injection	Completed
NCT04315025	UC-MSC	I/II	18	Peribulbar injection	Completed
NCT04224207	WJ-MSC	III	32	Subtenon injection	Completed
NCT03963154	hESC- derived	I/II	7	Subretinal implantation	Ongoing
RPE					
NCT03073733	RPC(jCell	II	84	Intravitreal Injection	Completed
NCT02464436	hRPC	I/II	29	Subretinal injection	Ongoing
NCT02320812	RPCjCell	I/II	28	Intravitreal injection	Completed
NCT02280135	BMSC	I	8	Intravitreal injection	Completed
NCT01736059	BMSC	I	15	Intravitreal injection	Ongoing
NCT01560715	BMSC	II	50	Intravitreal injection	Completed
NCT01531348	MSC	I	14	Intravitreal injection	Completed
NCT01068561	BMSC	I	5	Intravitreal injection	Completed
NCT04284293	CNS10-NPC	I	16	subretinal injection	Ongoing
NCT00063765	NTC-210(CNTF)	I	10	Intraocular implants	Ongoing
NCT04925687	CD34 +	I	4	Intravitreal injection	Completed
NCT00447993	NTC-210(CNTF)	II	67	Intraocular implants	Completed
NCT00447980	NTC-210(CNTF)	II	73	Intraocular implants	Completed
NCT04238858	a-PRP	N.A	48	subtenon injection	Completed
NCT01530659	NTC-210(CNTF)	II	22	Intraocular implants	Completed

*RPC*, Retinal progenitor cell; *HRPC*, Human retinal precursor cell; *BMSC*, Bone marrow derived stem cell; *MSC*, Mesenchymal stem cell; *UC-MSC*, Umbilical cord mesenchymal stem cell; *WJ-MSC*, Wharton’s jelly derived mesenchymal stem cell; *CNS10-NPC*, Neuronal precursor cells; *CNTF*, Ciliary neurotrophic factor; *a-PRP*, Autologous platelet rich plasma

Clinical studies have also been conducted using retinal sheets derived from human fetus and transplanted into patients [123, 124]. With the development of human iPSCs, these cells can also be differentiated into RO and retinal sheets enriched with photoreceptors [125, 126]. iPSC-derived photoreceptors eliminate the ethical concerns associated with using embryo-derived cells and provide a sufficient number of pure photoreceptors [127]. Assawachananont et al. derived ROs from both mouse ESCs and iPSCs and transplanted the retinal sheets subretinally into the rd1 mouse model. The grafted tissue developed a structured outer nuclear layer and formed synaptic connections with host bipolar cells [128]. Subsequent studies using the rd1 mouse revealed that the transplanted retinal sheet was responsive to light [129]. In 2018, human ESC-derived retinal sheets were transplanted into a mouse model of retinal degeneration (NOG-rd1-2J). The transplant was successful, resulting in the formation of an outer nuclear layer, with some grafted retinas demonstrating light responsiveness [130]. A study involving human ESC-derived retinal sheet transplantation in rats and monkeys showed similar results, with successful graft implantation and maturation into photoreceptors; however, determining visual responsiveness from the transplanted tissue was challenging [131]. To our knowledge, only one study has involved subretinal transplantation of clinically graded iPSC-derived ROs into two human patients with advanced stages of RP (jRCTa050200027). Over a two-year period, the grafts survived, showed a slight increase in retinal thickness, and— in one patient— exhibited improved response to full-field light stimuli [132]. Further optimization is necessary to improve the efficiency of regenerative cell therapy. Nonetheless, it is important to acknowledge that this approach has the potential to address all mutations associated with RP and other retinal degenerative diseases, regardless of disease stage. With ongoing research, this method is expected to become even more significant in the future.

Despite progress, stem cell transplantation for retinal degeneration faces challenges. Our understanding of stem cell proliferation and differentiation remains limited, requiring further research for improved therapies [133]. Safety concerns like tumor formation and immune rejection are significant, but techniques such as pre-inducing embryonic stem cells into neural progenitors and developing xeno-free or immunosuppressive methods might minimize the risk.

## Conclusions

Significant advancements have been made in understanding the molecular mechanisms underlying splicing-related RP and in developing innovative therapies. Although approaches like gene augmentation, antisense oligonucleotides, and stem cell transplantation show great promise, each faces challenges in delivery, safety, and long-term efficacy. Most clinical trials are still in initial phases but they are offering a hope for patients. Current therapeutic strategies focuse on enhancing PRPF31 expression, correction of splicing by ASO and cell replacement. The ongoing use of patient-derived models, such as ROs, along with genomic and transcriptomic analyses, will be essential for uncovering disease-specific mechanisms and enhancing personalized treatment strategies. Ultimately, improving our comprehension of splicing defects and refining translational tools will lead to the development of safe and effective therapies aimed at preserving or restoring vision in RP patients. This should go hand-in-hand with establishing biomarkers such as splicing signatures, circRNA patterns, and tri-snRNP protein abundance. This is essential for patient stratification, predicting disease onset, and monitoring treatment response. Finally, for treatment targeting specific pathogenic variants, we will have to identify schemes to finance development, testing, production and delivery of these treatments that are costly and target only small number of patients.

## Data Availability

Not applicable.
